# Mortality among rough sleepers, squatters, residents of homeless shelters or hotels and sofa-surfers: a pooled analysis of UK birth cohorts

**DOI:** 10.1093/ije/dyab253

**Published:** 2021-12-06

**Authors:** James White, Meg Fluharty, Rosa de Groot, Steven Bell, G David Batty

**Affiliations:** Centre for Trials Research, School of Medicine, Cardiff University, Cardiff, UK; Behavioural Science and Health, Institute of Epidemiology & Health Care, University College London, London, UK; Department of Donor Medicine Research—Donor Studies, Sanquin Research, Amsterdam, the Netherlands; and Department of Epidemiology and Data science, Amsterdam Public Health Research Institute, Amsterdam UMC, VU University, Amsterdam, The Netherlands; Department of Clinical Neurosciences, University of Cambridge, Cambridge, UK; Department of Epidemiology and Public Health, University College London, London, UK

**Keywords:** Homelessness, squatting, sofa-surfing, mortality, cohort study

## Abstract

**Background:**

Homelessness encompasses a wide spectrum of experience. Rough sleepers and people attending homeless shelters have been found to be at an increased risk of mortality. It is unclear whether risks are also elevated in those squatting, living temporarily in low-cost hotels or ‘sofa-surfing’ with friends or family members. This study examines mortality in a representative nationwide sample of people who have slept rough, squatted, lived in shelters or low-cost hotels and sofa-surfed.

**Methods:**

Using unpublished data from two national birth cohorts, namely the National Child Development Study and the 1970 British Birth Cohort study, Cox proportional-hazards models and random-effects meta-analyses were used to analyse associations between homelessness and different types of homeless experience (rough sleeping, squatting, staying in a homeless shelter or low-cost hotel, and sofa-surfing) and mortality.

**Results:**

Out of the 23 678 participants, 1444 (6.1%) reported having been homeless and 805 (3.4%) deaths occurred. Homelessness was associated with an increased risk of mortality [hazard ratio (HR) 1.68, 95% confidence interval (CI) 1.24–2.26]. Mortality risk was raised across the spectrum of homeless experience, from sleeping rough (HR 4.71, 95% CI 2.38–9.30), to squatting (HR 6.35, 95% CI 2.73–14.75), staying in a homeless shelter (HR 4.89, 95% CI 2.36–10.11), staying in a low-cost hotel (HR 3.38, 95% CI 1.30–8.79 through to sofa-surfing (HR 2.86, 95% CI 1.84–4.42). Associations remained after separate control for socio-economic status, mental health, substance use, accidents and assaults, and criminality.

**Conclusions:**

Mortality rates were raised across all types of homeless experience. This included squatting and sofa-surfing that have not previously been reported. Studies that have omitted the less severe, but more prevalent, use of low-cost hotels and sofa-surfing may have underestimated the impacts of homelessness on mortality.

Key Messages

- This study tests the associations of different types of homeless experience (rough sleeping, squatting, staying in a homeless shelter or low-cost hotel and sofa-surfing) with mortality using two national representative birth cohorts.
- All types of homeless experience were associated with an increased risk of mortality. Those for squatting and sofa-surfing have not previously been reported.
- Housing policies should be revised to reduce the use of housing in low-cost hotels.
- The scope of homelessness prevention should be expanded to also include less severe forms of homelessness.

## Introduction

Worldwide, it is estimated that nearly 1.6 billion people have inadequate shelter.^1^ Previous research on mortality among homeless individuals has focused primarily on the extreme end of the spectrum, with people sleeping rough or in homeless shelters. Relative to the general population, these groups experience a strikingly increased risk of mortality, with some studies indicating that mortality rates may be as high as 10 times those apparent in the general population.^2^^,^^3^ More often, however, homeless people are placed in short-term low-cost collective dwellings, such as a hostels (e.g. YMCA/YWCA) or single-occupancy low-cost hotels with shared access to bathroom facilities.^4^^,^^5^ Individuals with limited housing options may also choose to initially ‘sofa-surf’, living with family or friends temporarily. Whilst these insecurely housed groups are typically less disadvantaged than rough sleepers, the number of people sofa-surfing in the USA has been estimated to be 10,^6^ and in England 8, times higher^5^^,^^7^ than the number rough sleeping.

In view of the paucity of evidence on the risk of mortality at the less severe end of the homelessness experience, we compared mortality rates across the spectrum from rough sleeping to sofa-surfing to a general-population comparator who had not been homeless and sought to explain any associations using information on individuals’ socio-economic, lifestyle, substance-use and criminal-justice experiences.

## Methods

We used data from the 1958 Birth Cohort study (also known as the National Child Development Study) and the 1970 British Birth Cohort study. Described in detail elsewhere,^8^^,^^9^ these are ongoing, geographically representative, prospective birth-cohort studies with samples drawn from England, Scotland and Wales. The 1958 cohort was approved by the National Health Service Research Ethics committee. The 1970 cohort was approved by the London Central Research Ethics Committee. Written informed consent was given by the parents of study participants before the start of data collection. We adhered to the guidelines for STrengthening the Reporting of OBservational studies in Epidemiology (STROBE) in the reporting in this manuscript.^10^ The year of assessment for all variables is provided in Supplementary Table S1 (available as Supplementary data at *IJE* online).

### Assessment of homelessness

At age 33 years in the 1958 cohort and age 30 years in the 1970 cohort, participants were asked whether they had ever been homeless (since age 23 years in the 1958 cohort and age 16 years in the 1970 cohort). Homelessness was defined as having to move out of a residence and having nowhere permanent to live (excluding living with parents). Respondents answering positively were then asked where they stayed while they were looking for permanent accommodation. Multiple responses were permitted, with the options of rough sleeping, a hostel or night shelter for the homeless, squatting (unlawfully staying in an uninhabited building or settling on a piece of land), bed and breakfasts (low-cost hotels), sofa (couch)-surfing [staying with friend(s) or relative(s); herein called sofa-surfing] and ‘other’ places.

### Mortality ascertainment

Members of the 1958 cohort were followed up on six occasions over 35 years for all-cause mortality, from March 1981 (aged 23 years) until December 2016 (aged 58 years), whereas participants from the 1970 cohort were followed up five times over 27 years, from March 1986 (aged 16 years) until December 2014 (aged 44 years). Vital status was derived from official death certificates, information from the National Health Service Central Register or fieldwork and cohort-maintenance work (<1% of deaths).

### Covariates

Covariates were identified a priori. In the 1958 cohort, these were assessed at age 33 or 42 years and in the 1970 cohort at age 30 years. Socio-economic status was based on responses to enquiries about school-leaving age, educational qualifications and employment status. Mental health problems were ascertained based on specialist treatment for a psychiatric problem since age 16 years (1970 cohort only), plus psychological morbidity in both cohorts based on a score of ≥7 on the Rutter Malaise Inventory.^11^ Self-reports of accidents and assaults that occurred since 16 years old that required treatment by a physician were recorded. Substance-use assessments included the lifetime use of illicit drugs [cannabis, ecstasy, amphetamines, lysergic acid diethylamide (LSD), poppers, magic mushrooms, cocaine, temazepam, ketamine, crack, heroin, methadone, other], whether they were a daily smoker and whether they had an alcohol problem, defined as a score of ≥2 on the CAGE (*c*utting down, being *a*nnoyed by criticism, feeling *g*uilty and *e*ye-openers) questionnaire.^12^ Experiences with the criminal-justice system included instances of being arrested, formally cautioned or convicted in a criminal court. Cohort members then reported on other aspects of their lifestyle including participation in regular exercise; the frequency of consumption of fruit, vegetables and salad; and height and weight, measured directly by a nurse, to calculate body mass index (BMI).

### Analysis

A detailed description of attrition in both cohorts is provided elsewhere.^13^ The main reason for loss to follow-up was members moving and not subsequently being traced. Refusal rates across were relatively low (1958: 13.2%; 1970: 7.3%), deaths were higher in the 1958 cohort and emigration was rare (<2%). Of the 17 634 individuals originally recruited in the 1958 cohort, 12 477 (70.8%) provided information on exposure to homelessness plus covariates. In the 1970 cohort, the equivalent numbers were 16 571 and 11 201 (67.6%). Missing data per variable ranged from 0% to 32.5% in the 1958 cohort and 0% to 10.5% in the 1970 cohort. There were 19 247 participants (9436 in the 1958 cohort and 9811 in the 1970 cohort) with no missing exposure or covariate data that made up the complete data sample. The resulting imputed analytical sample had 23 678 participants (12 477 from the 1958 cohort and 11 201 from the 1970 cohort).

We computed missing exposure and covariate data using multiple imputation by chained equations to generate 20 data sets. The imputation model included all missing exposure and covariate data and the Nelson–Aalen estimate of the cumulative hazard of survival time to increase statistical power.^14^ To test differences in baseline characteristics by exposure to homelessness, we used a logistic-regression model. We used Cox proportional-hazards models to compute hazard ratios (HRs) with accompanying 95% confidence intervals (CIs) to summarize the association between homelessness and mortality separately for each cohort. We ascertained that the proportional-hazards assumption had not been violated in each cohort by inspecting the Schoenfeld residuals. Exposure to any form of homelessness was compared with a reference category of not having been homeless. Next, we compared exposure to each type of homelessness to the reference category of never having experienced that type of homelessness.

We did not find interactions between homelessness and sex in the association with mortality in either cohort, so data were aggregated and sex-adjusted. For each cohort, we adjusted HRs for sex (the basic model), then added socio-economic status, mental health problems, substance misuse, accidents and assaults, criminal-justice contacts, lifestyle and, lastly, all covariates combined. In analyses on exposure to different types of homelessness, we adjusted for other types then followed this sequence of adjustments. We then combined the estimates from each cohort using a random-effects meta-analysis, resulting in a common HR, and computed the *I*^2^ statistic to examine heterogeneity in these estimates.

To examine the influence of missing data, we reran the analysis on a complete data sample. To understand the influence of different groups of substances, we adjusted HRs for sex plus daily smoking, alcohol problems, individual illicit drugs and opioids (including heroin and methadone). All analyses were computed using Stata 16 (StataCorp, College Station, TX) and R (version 4.03).

## Results

In the pooled sample, 6.1% of study members (*n* = 1453) had experienced some form of homelessness (6.0% in the 1958 cohort and 6.3% in the 1970 cohort). Of those who had been homeless, 75.0% had sofa-surfed, 28.6% had stayed elsewhere, 23.6% had used a hostel or homeless shelter, 22.5% had stayed in bed and breakfasts/hotels, 22.1% had slept rough and 17.3% had squatted (see Supplementary Figure S1, available as Supplementary data at *IJE* online). In people who reported exposure to more than one type of homelessness, sofa-surfing plus the ‘other’ category was the most frequently reported combination.

Table 1 provides the characteristics of the study members who had been homeless relative to those who had not been homeless. People who had been homeless reported a markedly higher prevalence of mental health problems, treatments for assault, criminal-justice experiences and smoking compared with those who had not. Opioid use was between 5 and 10 times as common in the homeless group than in the not-homeless group (see Supplementary Table S2, available as Supplementary data at *IJE* online).

**Table 1:** Characteristics of participants according to homelessness status

	1958 cohort			1970 cohort		
	Homeless					
	No	Yes	*P*-value	No	Yes	*P*-value
No. of participants	11 726	751		10 499	702	
Deaths	5.3 (620)	7.2 (54)	0.03	1.1 (115)	2.3 (16)	0.006
Female	50.1 (5870)	49.5 (372)	0.78	50.9 (5344)	58.5 (411)	<0.001
Socio-economic status						
Unemployed	4.3 (501)	5.8 (44)	0.94	2.8 (294)	8.5 (60)	<0.001
Left school before age 16 years	0.8 (89)	2.1 (16)	<0.001	1.9 (199)	7.4 (52)	<0.001
No qualifications	11.8 (1384)	11.9 (90)	<0.001	64.0 (6719)	74.2 (521)	<0.001
Mental health						
Psychiatric morbidity (Malaise score ≥7)	11.8 (1384)	10.7 (81)	<0.001	11.6 (1218)	28.2 (198)	<0.001
Seen specialist for psychiatric problem^a^	–	−		23.7 (2488)	50.4 (354)	<0.001
Physical health						
Seen doctor after an accident	39.2 (4595)	46.4 (348)	0.001	53.9 (5659)	60.5 (425)	0.001
Seen doctor after a violent assault, mugging or sexual assault	4.0 (471)	10.4 (78)	<0.001	6.4 (672)	17.1 (120)	<0.001
Criminal-justice experience						
Arrested by police	3.8 (440)	8.0 (60)	<0.001	16.2 (1701)	35.6 (250)	<0.001
Formally cautioned by police	3.3 (385)	7.1 (53)	<0.001	13.6 (1428)	30.9 (217)	<0.001
Been found guilty in court	4.0 (469)	7.4 (56)	<0.001	12.3 (1291)	28.8 (202)	<0.001
Substance misuse						
Alcohol problem (CAGE score ≥2)	0.1 (11)	0.5 (4)	0.003	11.4 (1197)	22.9 (161)	<0.001
Smoke every day	29.9 (3505)	42.2 (317)	<0.001	27.5 (2887)	54.6 (383)	<0.001
Lifetime illicit drug use	38.2 (4475)	53.4 (401)	<0.001	53.0 (5564)	75.4 (529)	<0.001
Lifestyle						
Obese (BMI >30)	12.0 (1403)	8.0 (60)	0.006	14.0 (1470)	14.5 (102)	0.69
Regular exercise	78.2 (9172)	78.1 (587)	0.97	79.0 (8294)	76.9 (540)	0.19
Fruit more than once a day	52.6 (6167)	52.4 (394)	0.95	20.2 (2121)	17.7 (124)	0.10
Salad or raw vegetables once a day	9.0 (1053)	9.8 (73)	0.53	18.0 (1890)	19.5 (137)	0.31

Results are % (*N*) of participants unless stated otherwise.

a Information not collected in the 1958 cohort.

CAGE (*c*utting down, being *a*nnoyed by criticism, feeling *g*uilty and *e*ye-openers); BMI, body mass index.

**Figure 1.**
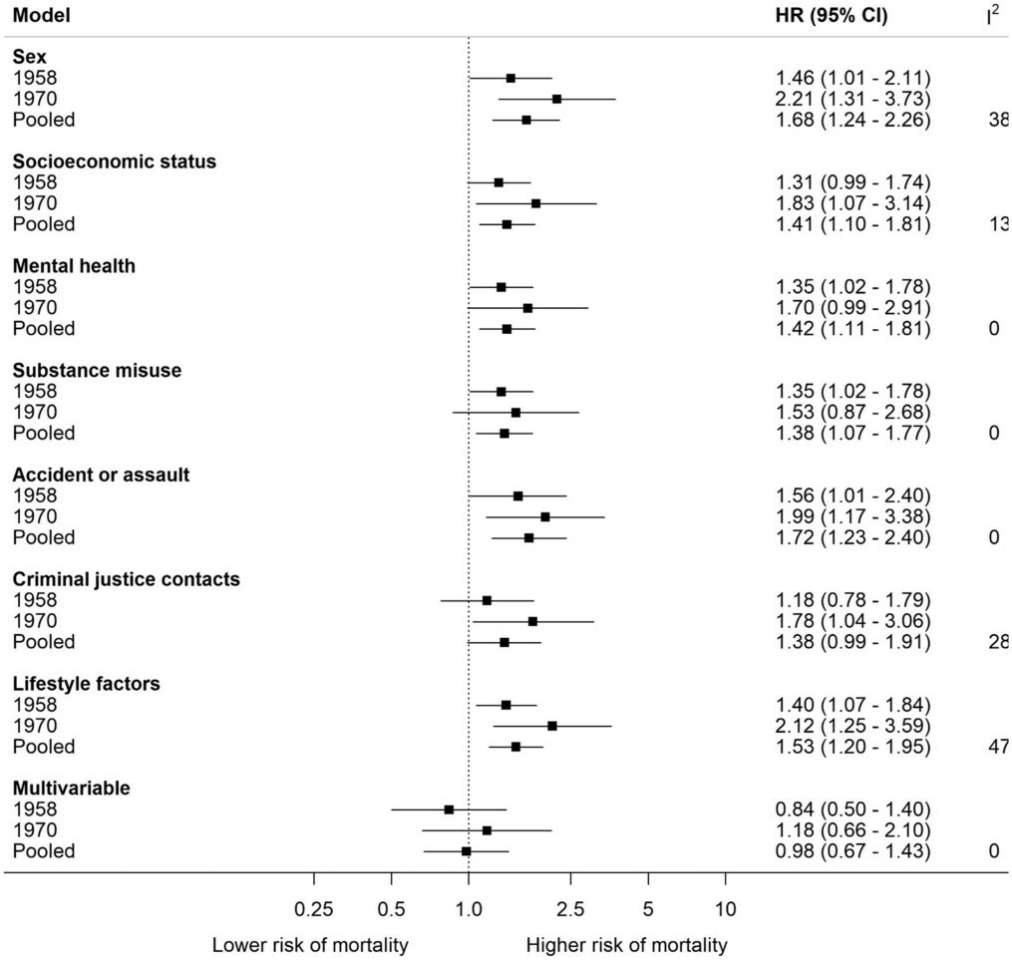
Association between homelessness and mortality (n = 23,678) Reference category is never been homeless. Multivariable model comprises all covariates above. HR: hazard ratio; CI: confidence interval

There were 805 deaths over a median follow-up of 27.2 years [interquartile range (IQR) 19.5–32.7] in the 1958 cohort and 8.7 years (IQR 3.8–11.2) in the 1970 cohort. Figure 1 shows the association between homelessness and mortality. After pooling and adjustment for sex, relative to people who had never been homeless, the experience of any type of homelessness was associated with excess mortality (HR 1.68, 95% CI 1.24–2.26). Relative to the sex-adjusted HRs for the association between homeless and mortality, additional adjustment for each set of covariates had a small attenuating effect and, after multivariable adjustment, the association was explained.

**Figure 2.**
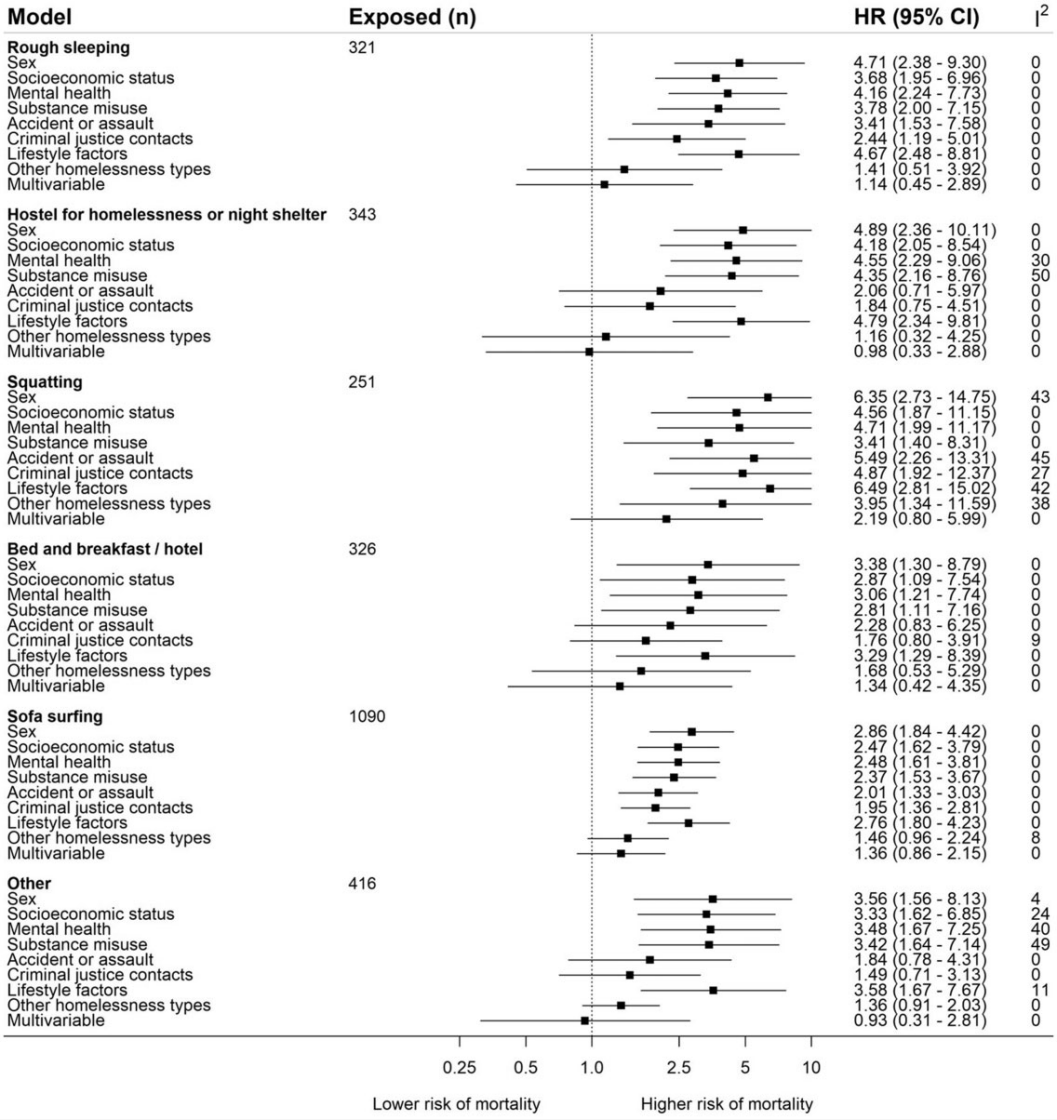
Association between type of homeless residence and mortality (n = 23,678) Reference category is not having experienced that type of homeless. Multivariable model comprises sex, mental and physical health, substance misuse, socioeconomic status, criminal justice experiences, and lifestyle. HR: hazard ratio; CI: confidence interval

Figure 2 reports the associations for the place where the study member stayed when homeless and mortality. The pooled sex-adjusted HR for squatting was 6.35 (95% CI 2.73–14.75), use of a homeless shelter was 4.89 (95% CI 2.36–10.11), rough sleeping was 4.71 (95% CI 2.38–9.30), bed and breakfast was 3.38 (95% CI 1.30–8.79), other places was 3.56 (95% CI 1.56–8.13) and sofa-surfing was 2.86 (95% CI 1.84–4.42). Some estimates were imprecise, as evidenced by the wide CIs. The *I*^2^ statistic was 0% for most HRs (range 0–50%). Adjustment for covariates had a small effect on the association between mortality and different types of homelessness. One exception was adjustment for other types of homelessness, which partially attenuated associations with mortality. We found little difference in the pattern of attenuation after separate adjustment for alcohol problems, daily smoking, any illicit drug and opioid use (see Supplementary Figure S2, available as Supplementary data at *IJE* online).

In the data sets in which there were no missing data, the CIs for estimates overlapped with those from the main results using imputed data, indicating that there were no meaningful differences (see Supplementary Figures S3–S5, available as Supplementary data at *IJE* online). The *I*^2^ statistic ranged between 53% and 79% for the analysis of homelessness, indicating heterogeneity in the HRs between cohorts. In the analysis of types of homelessness, the *I*^2^ statistic was 0% for most estimates, but heterogeneity was found for bed and breakfast (0–64%) and sofa-surfing (0–60%).

## Discussion

In this paper, we have reported for the first time the association between mortality and homelessness across the full spectrum of severity. Based on data from two representative national cohorts, mortality rates were higher across all categories of homeless experience than in a general-population comparator group that had not been homeless. In particular, the elevated risks of mortality among those squatting and sofa-surfing had not previously been reported.

Our results are consistent with previous findings of higher rates of mortality found in rough sleepers when compared with hostel users and to a general-population comparator group.^15^ Residents of hotels, motels and tourist homes have also been found to experience excess mortality when compared with people who are housed and in the lowest fifth of the distribution.^16^ Other studies on homelessness using economically matched comparator groups have reported conflicting results.^3^^,^^17^ We expanded the scope of homeless experience investigated and the risks reported suggest that these inconsistencies may be due to the wide range of homeless experience not captured in those data sets.

Our findings could be important in advancing understanding on factors that precipitate and perpetuate homelessness. Across a comprehensive range of explanatory factors, each led to a small reduction in the strength of the association between homelessness and mortality, but no one factor was particularly influential. There was some evidence that adjustment for substance misuse also had a slightly larger impact for those who had squatted relative to other types of accommodation. If this attenuation is consistent with mediation, interventions like improving security in hostels and access to effective treatments for substance use may help to mitigate the impacts of these forms of homelessness on mortality. Additional work examining cause-specific mortality according to the type of homeless accommodation may help to elucidate mechanisms underpinning these associations.

Homelessness is now increasing in the USA and the number of homeless individuals who are elderly is projected to triple by 2030.^18^^,^^19^ As part of the coronavirus 2019 pandemic response, many countries secured low-cost bed and breakfasts for homelessness people requiring isolation or quarantine for confirmed or suspected infection^20^ to facilitate social distancing^21^ and there was a national moratorium placed on evictions.^22^ In the UK, these initiatives were associated with a reduction in rough sleeping by around a third,^23^ with low-cost-hotel use increasing by 17%.^5^ These findings illuminate additional forms of homelessness such as squatting, which has hitherto not received as much attention in policy as being associated with premature mortality. There is increasing concern that once the moratorium on evictions and economic support is lifted, homelessness in all forms will increase.^24^ The presented results indicate that if these concerns are realized, life expectancy may reduce in these groups.

Limitations of our work include the impacts of loss to follow-up. We used multiple imputation to maximize the plausibility of the missing at random assumption and restore sample representativeness. Results were comparable when using the data sets with missing and imputed data, increasing confidence in the findings. Bias due to non-ignorable missing data cannot, however, be ruled out. As both cohorts used household tracking, homelessness people in the present analysis are likely to have a lower mortality risk than previous studies that sampled those who were currently homeless. This difference is likely to have attenuated our estimates of mortality risk towards the null. As exposure to homelessness, treatment for mental health problems and illicit drug use were retrospectively assessed, it is difficult to determine the temporal precedence of these events. It is therefore possible that these factors occurred before homelessness and that the attenuation that we found is more consistent with confounding than mediation. Conversely, in the 1958 cohort, criminal-justice experiences and illicit drug use were assessed at a subsequent wave to that at which homelessness was assessed. In this case, attenuation is more consistent with these factors acting as mediators of the homelessness–mortality relation.

## Conclusions

Our study provides evidence that exposure to any type of homelessness in early adult life can increase the risk of mortality. Mortality risk was shown to be raised across the spectrum of homeless experience. This included squatting and sofa-surfing, which had not previously been examined. These findings provide evidence that housing policy may need to be revised to reduce the use of housing in low-cost hotels and the scope of homelessness prevention expanded to include sofa-surfing.

## Ethics approval

The 1958 cohort was approved by the National Health Service Research Ethics committee. The 1970 cohort was approved by the London Central Research Ethics Committee. Written informed consent was given by the parents of the study participants before the start of data collection and conform to the Declaration of Helsinki.

## Author contributions

Concept and design: J.W. and G.D.B.; acquisition of data: M.F., J.W.; analysis: S.B., J.W., M.F., R.D.; interpretation of data: all authors; drafting of the manuscript: J.W.; critical revision of the manuscript for important intellectual content: all authors; final approval of the version to be published: all authors; agreement to be accountable for all aspects of the work thereby ensuring that questions related to the accuracy or integrity of any part of the work are appropriately investigated and resolved: all authors.

## Data availability

Data from the National Child Development Study and 1970 British Cohort Study are available to researchers upon application (https://discover.ukdataservice.ac.uk/).

## Supplementary data

Supplementary data are available at *IJE* online.

## Funding

This work was supported by the Centre for the Development and Evaluation of Complex Interventions for Public Health Improvement (DECIPHer), a UKCRC Public Health Research Centre of Excellence. Joint funding [MR/KO232331/1] came from the British Heart Foundation, Cancer Research UK, Economic and Social Research Council, Medical Research Council, the Welsh Government and the Wellcome Trust, under the auspices of the UK Clinical Research Collaboration (J.W.). G.D.B. is supported by the UK Medical Research Council [MR/P023444/1] and the US National Institute on Aging [1R56AG052519-01; 1R01AG052519-01A1]. S.B. is supported by the British Heart Foundation [RG/4/32218]. M.F. is supported by the Economic and Social Research Council [ES/K000357/1] and Academy of Medical Sciences/the Wellcome Trust ‘Springboard Health of the Public in 2040’ Award [HOP001\1025]. No funding bodies had any role in the study design, data collection and analysis, decision to publish or preparation of the manuscript.

## Conflict of interest

None declared.

## Supplementary Material

dyab253_Supplementary_Data
