# Supplementary material for: Mortality among rough sleepers, squatters, residents of homeless shelters or hotels and sofa-surfers: a pooled analysis of UK birth cohorts
Source: Int J Epidemiol. 2021 Dec 6;51(3):839–46. doi: 10.1093/ije/dyab253 (PMC9189982; doi:10.1093/ije/dyab253)
Supplement: Supplementary file 1 [file dyab253_supplementary_data.zip › ije-2021-07-1036-File005.odt]

Supplementary file

- Table S1. Year of assessment of study variables

- Table S2. Drug Use among Participants According to Whether they have been Homeless (n = 23,678)

- Figure S1. Type of Homeless Residence in the 1958 National Child Development Study and the 1970 British Birth Cohort Study

- Figure S2. Association between Homelessness and All-cause Mortality after Adjusting for Different Substances (n = 23,678)

- Figure S3. Association between Homelessness and All-cause Mortality in Participants with No Missing Data (n = 19,247)

- Figure S4. Association between Type of Residence and All-cause Mortality in Participants with No Missing Data (n = 19,247)

- Figure S5. Association between Homelessness and All-cause Mortality after adjusting for Different Substances in Participants with No Missing Data (n = 19,247)

83.8

39.4

33.4

31.6

27.9

26.7

65.6

17.0

10.0

12.7

18.9

7.1

0

20

40

60

80

Sofa surfing

Other place

Rough sleeping

Bed and breakfasts/ hotels

Hostel or homeless shelter

Squatted

1958 cohort

1970 cohort

%

20

40

60

80

Figure S1. Type of homeless residence in the 1958 National Child Development Study and the 1970 British Birth Cohort Study

Table S1. Year of assessment of study variables

| Variables | 1958 cohort | 1970 cohort |
| --- | --- | --- |
| Homelessness (exposure) | 1991 | 2000 |
| Mortality (outcome) | 2016 | 2014 |
| Covariates |  |  |
| Gender | 1991 | 2000 |
| Socioeconomic status |  |  |
| Unemployed | 1991 | 2000 |
| Left school before 16-years | 1991 | 2000 |
| No qualifications | 1991 | 2000 |
| Mental health |  |  |
| Psychiatric morbidity (Malaise score ≥7) | 1991 | 2000 |
| Seen specialist for psychiatric problem | Not collected | 2000 |
| Physical health |  |  |
| Seen doctor after an accident | 1991 | 2000 |
| Seen doctor after a violent assault mugging or sexual assault | 1991 | 2000 |
| Criminal justice experience |  |  |
| Arrested by police | 2000 | 2000 |
| Formally cautioned by police | 2000 | 2000 |
| Been found guilty in court | 2000 | 2000 |
| Substance misuse |  |  |
| Alcohol problem (CAGE score ≥2) | 1991 | 2000 |
| Smoke every day | 1991 | 2000 |
| Lifetime illicit drug use | 2000 | 2000 |
| Lifestyle |  |  |
| Obese (BMI >30) | 1991 | 2000 |
| Regular exercise | 1991 | 2000 |
| Fruit more than once a day | 1991 | 2000 |
| Salad or raw vegetables once a day | 1991 | 2000 |

Table S2. Drug use among participants according to whether they have been homeless (n = 23,678)

|  | Homeless |
| --- | --- |
|  | 1958 cohort | 1970 cohort |
|  | No | Yes | P-value | No | Yes | P-value |
| Lifetime illicit drug use |  |  |  |  |  |  |
| Cannabis | 28.0 (3283) | 52.1 (392) | <0.001 | 49.1 (5,122) | 71.7 (552) | <0.001 |
| Ecstasy | 2.1 (247) | 6.1 (46) | <0.001 | 14.6 (1,522) | 33.3 (256) | <0.001 |
| Amphetamines | 6.9 (814) | 21.2 (159) | <0.001 | 20.9 (2,180) | 44.6 (343) | <0.001 |
| LSD | 4.0 (468) | 13.9 (104) | <0.001 | 12.6 (1,314) | 32.0 (246) | <0.001 |
| Poppers | 4.1 (481) | 13.5 (102) | <0.001 | 19.2 (2,002) | 35.8 (276) | <0.001 |
| Magic mushrooms | 5.3 (624) | 20.2 (152) | <0.001 | 12.9 (1,346) | 31.3 (241) | <0.001 |
| Cocaine | 4.0 (465) | 11.7 (88) | <0.001 | 12.6 (1,314) | 27.3 (200) | <0.001 |
| Temazepam | 3.2 (375) | 8.5 (64) | <0.001 | 4.4 (461) | 16.8 (129) | <0.001 |
| Ketamine | 0.3 (39) | 0.6 (4) | 0.40 | 1.9 (198) | 9.2 (71) | <0.001 |
| Crack | 0.5 (57) | 1.1 (8) | 0.06 | 1.3 (136) | 8.6 (66) | <0.001 |
| Heroin | 0.8 (95) | 4.6 (35) | <0.001 | 1.2 (125) | 9.6 (74) | <0.001 |
| Methadone | 0.6 (65) | 1.5 (11) | 0.01 | 0.1 (10) | 6.3 (49) | <0.001 |
| Other drug a | .. | .. | .. | 3.0 (313) | 8.5 (65) | <0.001 |

Results are % (N) of participants unless stated otherwise. Data collected at 33 and 42-years in the 1958 and 30-years of age in the 1970 cohort. 

a Information not collected in the 1958 cohort.  LSD = Lysergic acid diethylamide

A picture containing text, receipt

Description automatically generated

Figure S2. Association between homelessness and mortality after adjusting for different substances (n = 23,678)

Reference category is never been homeless. Smoking (daily smoker or not), having a possible alcohol problem (CAGE score ≥ 2) and ever having used illicit drugs (cannabis, ecstasy, amphetamines, LSD, poppers, magic mushrooms, cocaine, temazepam, ketamine, crack, heroin, methadone, other). 

HR: hazard ratio; CI: confidence interval.

A picture containing text, receipt

Description automatically generated

Figure S3. Association between homelessness and mortality in participants with no missing data (n = 19,247)

Reference category is never been homeless. Multivariable model comprises all covariates above. 

HR: hazard ratio; CI: confidence interval

Diagram, schematic

Description automatically generatedFigure S4. Association between type of residence and mortality in participants with no missing data (n = 19,247)

Reference category is never been homeless. Multivariable model comprises all covariates above. 

HR: hazard ratio; CI: confidence interval 

A picture containing text, receipt

Description automatically generated

Figure S5. Association between homelessness and mortality after adjusting for different substances in participants with no missing data (n = 19,247)

Reference category is never been homeless. Smoking (daily smoker or not), having a possible alcohol problem (CAGE score ≥2) and ever having used illicit drugs (cannabis, ecstasy, amphetamines, LSD, poppers, magic mushrooms, cocaine, temazepam, ketamine, crack, heroin, methadone, other). 

HR: hazard ratio; CI: confidence interval.
